# Understanding the Barriers and Opportunities for Effective Management of Shared Sanitation in Low-Income Settlements—The Case of Kumasi, Ghana

**DOI:** 10.3390/ijerph17124528

**Published:** 2020-06-23

**Authors:** Prince Antwi-Agyei, Bismark Dwumfour-Asare, Kwaku Amaning Adjei, Raphael Kweyu, Sheillah Simiyu

**Affiliations:** 1 Department of Civil and Environmental Engineering, University of Energy and Natural Resources (UENR), Regional Centre of Energy and Environmental Sustainability (RCEES), P.O. Box 214, Sunyani BS0061, Ghana; 2 Department of Environmental Health & Sanitation Education, University of Education Winneba, Asante-Mampong Campus, P.O. Box 40, Asante-Mampong AM0013, Ghana; dwumfourasare@gmail.com; 3 Department of Civil Engineering, Regional Water and Environmental Sanitation Centre (RWESCK), Kwame Nkrumah University of Science and Technology (KNUST), PMB, University Post Office, Kumasi AK448, Ghana; kaadjei.soe@knust.edu.gh; 4 Department of Geography, Kenyatta University, P.O. Box 43844, Nairobi 00100, Kenya; rufmulaa@gmail.com; 5 Urbanisation and Well-being Unit, African Population & Health Research Center, P.O. Box 10787, Nairobi 00100, Kenya; ssimiyu@aphrc.org

**Keywords:** barriers, behaviour change, compound, O&M, shared toilet, Ghana

## Abstract

Improved sanitation for all is a daunting task for low-income countries, and shared toilets often provide an alternative to private household sanitation for most urban residents. This study sought to provide better understanding of the existing barriers and opportunities for improved management of shared sanitation. The study used focus group discussions and in-depth interviews with 70 users (landlords and tenants) of shared sanitation in Kumasi, Ghana to assess barriers and opportunities of “high-quality” shared sanitation. The commonly used toilet facilities were dry toilets—Kumasi Ventilated Improved Pit latrine and Ventilated Improved Pit latrines; and flush systems—water closet and pour flush connected to septic tanks. Between 2 and 21 households, or 4 and 84 people, shared one facility. Participants’ description of “high-quality” (Ideal) shared sanitation was centred on cleanliness, user behaviour, smell, and user crowding. They also identified challenges of shared sanitation as overcrowded users, poor user behaviours, conflicts among users, and high cost associated with frequent desludging. However, opportunities for improvement included users’ preference for shared toilets due to enjoyed benefits, existing facility management practices, and mutual understanding among users (tenants and landlords). Interventions and policy guidelines to influence behaviour change of shared sanitation users are proposed and are intended to be delivered by local government and users.

## 1. Introduction

Access to adequate sanitation has both immediate and long-term benefits for socioeconomic impact. It may improve health by reducing diarrhoea and gastrointestinal diseases [1] and/or protect the environment for socio-economic development and poverty reduction [2]. Yet, approximately 673 million people practice open defaecation, and the situation is worse in Southern Asia and Sub-Saharan Africa [3]. The majority of people in low- and middle-income countries rely on onsite sanitation facilities shared by more than one household. In Ghana, for instance, shared sanitation is commonplace, and a large proportion of residents in low income areas live in compound houses, serving over 50% of the population [3,4] due to factors such as financial constraints and lack of space. Meanwhile, the Joint Monitoring Program (JMP) of WHO/UNICEF classifies shared sanitation as “limited” (the use of improved sanitation facilities which are shared with other households) service because of concerns that they are often very dirty and unhygienic, mainly due to poor behaviour and practices of users, which are also linked to poor operation and maintenance arrangements [5,6]. Shared toilets have also been found to expose users to high health risks, such as sanitation-related diseases including diarrhoea, gastroenteritis, and hookworm [7,8]. Because of the above concerns, limited sanitation is not included as an indicator in the monitoring of Sustainable Development Goal 6 (SDG 6) targets, but rather basic sanitation (the use of improved sanitation services that are not shared with households).

Shared (“limited”) sanitation, however, serves most people in low-income settlements and, in most cases, it is the only solution to their sanitation needs. Thus, excluding shared sanitation as an SDG 6 indicator could serve as a disincentive for funding and other support, and prevent the need to legitimise shared maintenance schemes and upgrading of large numbers of existing shared toilets to acceptable standards [9,10]. Consequently, underfunded shared sanitation could deprive most people access to this sanitation option, which may be the only alternative for crowded and low-income urban areas, serving to reduce and/or eliminate open defaecation [7] and prevent most countries in the developing world from meeting the targets in the SDG. While most studies have focused on quantitative determinants of the quality of shared sanitation, other studies have also focused on investigating the health outcomes between shared sanitation, household sanitation and compound sanitation [7,11,12], and development of behaviour change interventions to improve shared sanitation [13]. For example, some studies assert that toilets shared by fewer households can be considered improved (high quality) sanitation [6,14] but others argue that any amount of sharing still has negative outcomes on health [4]. Meanwhile, quality of shared sanitation is linked to user management practices including users’ cooperation, collective decision making, social norms, and landlord–tenant relationships [6]. Studies in Kenya suggest that a good landlord–tenant relationship is often linked to productive management practices by all toilet users, which are also associated with adequate shared sanitation, as well as proper toilet cleanliness and maintenance [6,15].

While the outcomes of previous studies are important and have contributed to the agenda of shared sanitation, there is still little evidence on the qualitative assessment and, in particular, on the indicators that users attribute to proper management of shared sanitation. There is also limited evidence related to the operation and maintenance arrangements as one of the quality typologies of shared sanitation, which have been the basis for the exclusion of shared sanitation from the SDG 6 indicators. This paper attempts to fill this knowledge gap by using Kumasi, Ghana as a case study. The study emphasises the need for an in-depth understanding of the barriers and opportunities for improving the quality and management of shared sanitation that are necessary for inclusive policies and interventions. The study forms part of a two-country project, the RESSA study—Research on shared sanitation in Africa—funded under the Leading Integrated Research for Agenda (LIRA) 2030 agenda, which seeks to understand the management of “high-quality” shared sanitation facilities in low-income settlements in Kenya and Ghana. There are several typologies of shared sanitation based on their location, accessibility, ownership, user group size, user group restrictions, management, payments, and operation and maintenance arrangement, among other factors [9,16]. This study, however, excludes communal or public toilets, which are often open to all community and non-community members, including transient populations. The study focuses on household shared sanitation facilities that are often used by a limited number of households on the same compound and who often know each other.

### Theoretical Approach

The Behaviour Change Wheel (BCW) framework was adapted for this study to help identify user behaviour that needs to be improved in relation to management of shared sanitation. BCW generally facilitates implementation of behaviour change interventions through precise and simultaneous targeting of multilevel systems [17,18,19]. The strength of BCW lies in its diagnostic potency at all levels (individual, community, and population, as well as their interactions) to identify target behaviour sources and factors [18]. The BCW framework has been applied by the International Red Cross in health training [18] and it has found several applications in the UK and other countries (e.g., India, Kenya, Thailand, USA, and the Netherlands) [18,20].

Application of the BCW begins with characterisation of behaviour and its interconnected systems, thus classifying behaviour change intervention activities [18,20]. At the core of BCW is the COM-B system, which postulates that behaviour occurs as interaction of three elemental conditions: (1) Capability (C)—a psychological and physical ability including strength, skills, knowledge and capacity for understanding, to engage in or enact certain behavioural activities; (2) Opportunity (O)—the factors of resources, locations, physical barriers, concepts available in language, exposure to ideas, among others, that enable or prompt behaviour; and (3) Motivation (M)—the reflective and automatic mechanisms that energise and direct (activate or inhibit) behaviour, not just goals and conscious decision-making, but also brain processes such as planning, habits, emotional reactions, drives, and analytical decision-making [18,20].

In addition, BCW adoption requires answering pertinent questions such as “Why are behaviours as they are, what needs to change, who needs to do what, when, where, and how?” [18]. The data from target populations, especially for COM-B diagnosis, often involves qualitative structured interviews [21]. Once challenging or problematic behaviours are identified for interventions, it is important that the final interventions are translated into specific Behaviour Change Techniques (BCTs) [18,21]. BCTs are “the smallest components of behaviour change interventions that on their own in favourable circumstances can bring about change” [19]. In this study, the core of BCW, COM-B, is used to diagnose and analyse barriers to positive behaviour associated with tenants and landlords using compound shared toilet facilities in Kumasi.

## 2. Methods

### 2.1. Study Area

Kumasi is the second largest city in Ghana and the capital of the Ashanti Region. It is located about 270 km north west of the national capital, Accra, and covers a total land surface area of about 250 square kilometres [22]. The city has a population of about 3.3 million people with an annual growth rate of about 4.4% as of 2020 [23]. The average household size is about 4 [22]. Over the years, the city has seen the growth and expansion of slum and other informal settlements. Access to sanitation is a major challenge in the city, as about 36% of the city’s population rely on public toilets, while 2.4% have no toilet facility [22]. About half of the population in Kumasi also live in informal settlements, which are often characterised by poor water supply, sanitation and hygiene. Less than 10% of Kumasi’s households are connected to a sewer network. Sewerage networks connected to simple treatment plants are available in 3 communities [22]. About 70% of Kumasi’s population lives in rental properties, mostly in multi-storey or compound houses accommodating 5 to 20 families. One or two toilets may be available, shared by all families or exclusively used by the landlord [24].

To achieve some heterogeneity in the study areas, the study was undertaken in three suburbs of Kumasi—Ayigya, Ahwiam, and Oforikrom. These suburbs, located along the main Accra–Kumasi road, are typically characterised by poor housing, poor access to services, and poor sanitation [25]. Most of the housing in the settlements comprises single storey traditional compound houses that are inhabited by more than one household [26]. Sanitation facilities within the settlements are mostly shared among the compound members [27].

### 2.2. Study Design and Data Collection

The study was cross-sectional based on a transdisciplinary approach. This iterative approach started with the formation of a joint research team involving stakeholders from academia, local government institutions, community representatives and implementation agencies. The group collaboratively framed the research problem, which was identified as “poor management of shared sanitation facilities”; the group is expected to continue the engagement, which will lead to co-production of solution-oriented and transferable knowledge. This study, however, focuses on the qualitative research approach employed to help understand the factors influencing the identified problem.

Field data collection spanned a period of one month in 2019. Focus group discussions (FGDs) and in-depth interviews (IDIs) were used to investigate factors at the compound level that potentially influence effective management and, in part, the quality of shared facilities as perceived by users. The FGD and IDI guides were developed to capture data and information on key themes, including existing benefits, challenges, cleanliness behaviours, practices and norms (positive and negative) associated with the use and management of shared sanitation, and the perceived barriers and inherent opportunities that can be harnessed to improve the management of shared sanitation. The full details of FGD and IDI guides are provided as supplementary material to this paper.

The sample sizes for these two data collection approaches (FGD and IDI) were reached based on the principle of saturation (a point where no new information was being made available) [28]. The selection of compounds and respondents was purposive, based on the premise that the respondents shared a toilet with at least one other household on the compound in a low-income community. Environmental health officers (EHOs) at the Oforikrom Municipal Assembly (local government), which has oversight responsibility of the study areas, assisted in the identification and selection of participants for FGDs. We relied on the EHOs due to their existing sanitation monitoring activities, their already established relationship with community leaders, and their knowledge of community dynamics. Actual interviews for the FGDs were conducted by the researchers themselves. In the case of IDIs, trained enumerators visited compound houses and interviewed only one household on that compound upon receipt of consent. As much as possible, enumerators tried to balance the number of tenants and landlords, as well as males and females, but this depended on the availability of who was present at the time of the visit.

Again, female and male participants (landlords and tenants) were interviewed to help understand the perspectives of these different user groups, their requirements, and the different responsibilities (especially men and women) regarding management of shared sanitation facilities. All face-to-face interviews were conducted at the respondents’ homes. For FGDs, the different groups (landlords and tenants) included men only, women only, and a mixed group of men and women. FGDs were held at vantage points selected by the EHOs in collaboration with community leaders. In-depth interviews took between 30 and 45 min, while FGDs took an average of 60 min. Both were audio recorded and complemented by detailed notes.

#### Ethical Considerations

The full information sheet (containing details of the study) was explained to participants before consent was taken for the interviews—individual written consent in the case of the IDIs and group oral consent for the FGDs.

The protocol for this study received ethical approval from the Centre for Scientific and Industrial Research (CSIR) in Ghana, with Ref: CSIR/IRB/PI/VOL1 in October 2018.

### 2.3. Data Management and Analysis

The specific behaviours associated with shared sanitation management among participants were assessed by analysing their prevalence as done in behavioural studies [20]. Audio recordings of interviews were transcribed following the approach recommended by Barker et al. [21]. Transcripts were analysed after coding, for prevalence of specific context behaviour using inductive thematic and content analysis of the main themes and salient categories [29,30,31] with the aid of Microsoft office (Word and Excel). In addition, the number of times coded topical responses emerged was counted as part of the prevalence analysis [32]. Codes used were key terms (i.e., cleanliness, benefit, crowding of users, etc) that formed the main theme of questions in the interview guides. Responses whose content represented the codes were assigned accordingly [32]. The final analyses focused on main themes, including users’ descriptions of the factors that could improve the quality of their shared toilet (herein referred to as “high quality” shared toilet); reasons for sharing the toilet; operations and maintenance activities; and behaviour of toilet users. Four other researchers reviewed the analysis for discrepancies in deductions and judgement, and synchronisation for better understanding of the main findings and emerging concepts. The analysis also adopted the use of the COM-B model to help identify the behavioural determinants (barriers and opportunities) of quality shared sanitation emerging from the main themes [21,33].

### 2.4. Limitations of the Study

This study was largely based on qualitative data and depended on self-reported data given by selected study participants. The study relied on purposive sample of participants from compound houses that shared toilet facilities at least among households in low-income urban settlements. Thus, not all landlords and tenants in all low-income settlements within Kumasi had equal chance of being selected, and hence the findings are only indicative of the prevailing situation in the study areas, and likely of other areas with similar settings.

## 3. Results and Discussion

### 3.1. Characteristics of Participants

**Figure 1:**
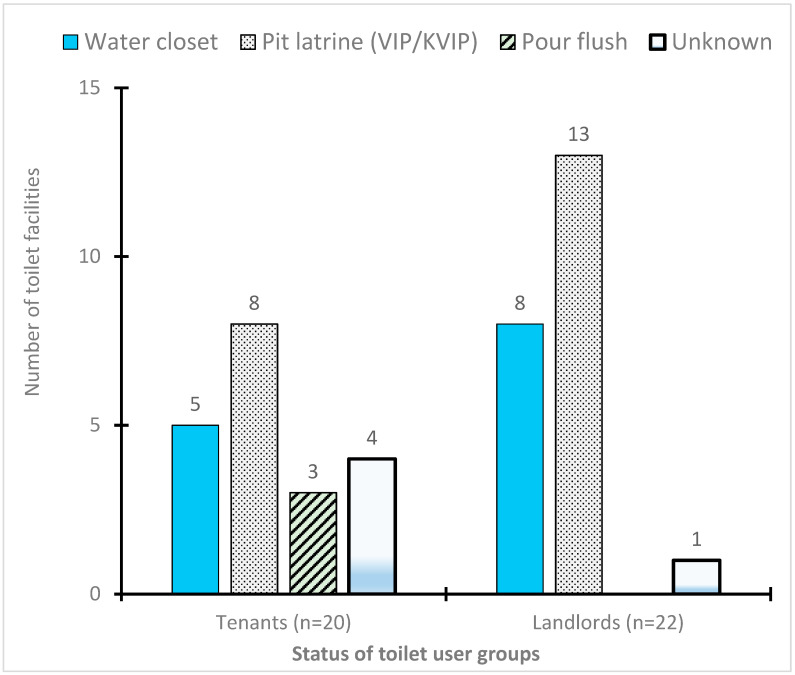
Types of shared sanitation facilities used by participants of the in-depth interviews (IDI). KVIP—Kumasi ventilated improved pit latrine, VIP—ventilated improved pit latrine.

All participants in the study were adults aged above 20 years. The total number of participants were 70, comprising 28 people from four FGDs, and 42 from IDIs (Table 1). There were more female participants than males (67% vs. 33%); this was preferred because issues around household sanitation are considered as part of women’s responsibilities. Similarly, landlords were also represented more than tenants (53% vs. 47%) although equal numbers were expected. The total number of occupants in each of the compound houses sharing toilet facilities from the FGDs was between 10 and 84 persons. The higher numbers came from bigger compounds, including multi-storey buildings with tenement housing, which is characteristic of urban areas in Kumasi [34,35]. In addition, FGDs and IDIs revealed that most landlords usually stayed on the same compound with their tenants, and compound occupancy could range from 2 to 19 households (also rooms), which aligns with an earlier study that reported 10–20 rooms [35]. The toilet types used by participants (FGDs and IDIs) were pit latrines in the form of Kumasi ventilated improved pit (KVIP) and Ventilated improved pit (VIP), water closet, and pour flush connected to septic tanks (Figure 1), and these were similar to toilet options reported in other studies [36,37]. Generally, most respondents shared toilets because of the high number of occupants in the compounds, and this confirms a similar study that found a strong association between high compound occupancy and the use of shared sanitation as the main sanitation service level in Ghana [38].

### 3.2. Reasons, Benefits and Challenges Associated with Sharing Sanitation Facilities

Participants reported that the use of compound toilets is a right enjoyed by users as a result of paying rent and this has been confirmed in other studies [39]. Participants claimed that the majority of households use the available compound toilets. However, one out of five households will use nearby public toilets instead of the compound toilets. According to participants, the very few tenants (none among study participants) who refuse to use compound toilets have reasons including personal issues (undisclosed), and unwillingness to participate in toilet cleaning chores and to pay for operations and maintenance costs (especially desludging). Meanwhile, public toilets in Kumasi are noted to be associated with poor cleanliness and hygiene issues [35,40] and these tenants’ preference for them could be partly to avoid responsibilities enumerated earlier. As reasons why users are unwilling to partake in cleaning shared toilets, similar studies in Uganda and Kenya identified lack of cleaning materials or detergents, the feeling that nobody was responsible for cleaning them, the ever-dirty state of the shared toilets, and poor user behaviour, such as soiling the toilet or improper cleaning [6,8].

“*… most of the tenants use the toilet but others don’t because they don’t want to clean it.*”

(Landlady, FGD)

However, two participants from the IDI shared key experiences of their co-tenants: such tenants use public toilets instead to 1) avoid paying an extra GHS 100 (i.e., a one-time payment of USD 18) on top of the two-year rent (an unusual compound tenancy condition); and 2) avoid quarrelling with users who have poor toilet use behaviour. In the first case, tenants realised that some landlords use compound toilets as extra privileges to overprice rental accommodation and felt cheated by landlords. Meanwhile, other tenants saw the extra fee to use their compound toilets to be cheaper than having to pay to use a public toilet over the same period.

“*…I continue to share the toilet because I have paid as part of my rent already. I paid two years advance rent, and I don’t need to spend anything extra on public toilet for two years. So, the toilet being here has helped me to save a lot of money and it is also convenient since you can use it at any time.*”

(Male tenant, IDI)

“*… because we are many using one toilet, you may fight with people over the use of the facility. Because we don’t have water in the house, some people barely use enough water to flush the toilet leaving it dirty. Some too will use kitchen wastewater to flush toilet after use. These are some of the reasons I am not using the toilet facility.*”

(Female tenant and caretaker, IDI)

Meanwhile, the few participants with landlord-tenant separated toilets made these comments:

“*There are two toilets in the house now because the landlord has added one solely for his household when they moved in to stay here with us.*” (Male tenant, IDI). “*…the landlady herself does not live here, it is her daughter (caretaker) and family who have separate toilet without sharing with tenants.*” (Female tenant, IDI). “*…There are two cubicles, but one is for the tenants and the other is for the landlady and her family alone.*”

(Female tenant, IDI)

Also, only one out of 42 participants (IDIs) indicated that their compound toilets were gender-separated, that is, two facilities or cubicles, one each for male and female users (Table 1).

In the current study, crowding of users around toilet facilities is better represented using findings from IDIs because more participants were able to give some numbers. An average of 16 users could be found sharing a compound toilet (around 15 and 17 users per facility for tenant and landlord groups respectively, Table 1). Compounds with two toilet facilities were found among only seven out of 70 participants (10%), which existed as gender-separated (two of 70) and/or landlord–tenant separated (five of 70). The proportion of landlord–tenant separated toilets in this study is lower than the 12% reported by Mazeau et al. [39] in a similar study in Kumasi. Meanwhile, our findings show a higher toilet-user crowding than an earlier study in Kumasi with eight households per facility [36], and also the 30 users per facility recommended earlier by the WHO/UNICEF Joint Monitoring Program (JMP) [41]. Based on the number of households, user crowding in this study is also higher than the three and five households per facility proposed by the Water and Sanitation for Urban Poor (WSUP) and WHO/UNICEF JMP as appropriate shared sanitation [41,42], though the recommendation by the WHO/UNICEF lasted only a year without any explanation [43]. Meanwhile, in Ghana the existing regulations, including local authority by-laws, are not explicit on toilet user capacity for household sanitation facilities, except in the case of chemical toilets in the building regulation, where the number of users is expected to be eight to 10 persons [44], likely for two to three households.

Almost all compound toilets used by participants were overcrowded, thus explaining some of their current concerns. For instance, there were strong complaints of long waiting-times for users to take turns due to queues, especially in the morning and in houses with many children (children were largely accused of making toilets dirty). There were also complaints of a large number of users with poor behaviour contributing to untidy toilet conditions.

“*…for my house the people are many, you may make the place neat, but another person may make the place dirty unless you always clean it yourself. That is how it is when many people are sharing a toilet*”

(Landlady, FGD)

“*…sometimes you may need to use the place urgently, but you find that someone is there already, and the person is keeping long there, which can be very uncomfortable.*”

(Landlady, FGD)

Almost all participants indicated that the compound toilets, regardless of their challenges, have been helpful. Some of the benefits to participants are reported in Table 2.

Participants commonly gave responses that could be categorised into the following four main themes on benefits, which are ranked from the highest to the lowest in terms of the number of times mentioned in IDIs:

- accessible and provide convenience at any time including night hours and emergencies;
- provides time savings (toilet within compound, avoids long distances to public toilets);
- provides safety and security compared to going outside to use public toilets; and
- provides cost savings compared to using public toilets.

In addition to this list, three other benefits were reported with one common to both landlords and tenants, namely, that compound toilets confer some dignity and pride. The landlords added that compound toilets could contribute to ending open defecation and spread of disease, and minimise distress commonly associated with long queues at public toilets (Table 2). These benefits are similar to those reported in other studies [45,46]. In addition, the benefits enjoyed by participants emphasise the importance of compound shared toilets as practical alternatives to inadequate household sanitation services in low-income urban settings [6]. This could also explain why almost one-third of Ghanaians would opt for shared toilets due to private ownership barriers, including insecure land tenure, lack of funds and bio-physical factors [10].

**Figure 2:**
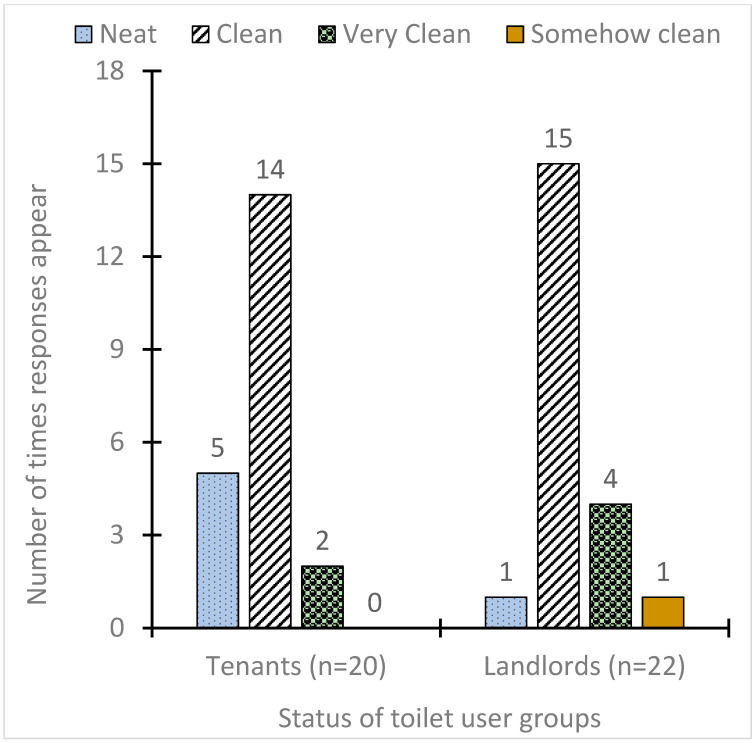
Perception of users regarding compound toilet cleanliness.

It can be hypothesised that participants generally perceived compound toilets to be cleaner than public toilets because almost all participants reported that their toilets were clean or better (Figure 2). This perception of participants on the cleanliness of their compound toilets contradicts the well-known assertion that shared toilets are often dirty [6,42,47]. The contradiction could be due to the fact that users claim more commitment to keeping the toilet clean, and comparatively less crowding, unlike the reverse in most other cases, which strongly decreases the cleanliness of shared toilets [6,48]. Aluko et al. [48], in a study in Tanzania, reported that it is possible to find the majority of shared toilets clean when there are positive attitudes towards cleanliness and maintenance.

Only two tenants reported no benefits from their compound toilets because of poor hygienic conditions making the toilets unusable as expected—“*…Our toilet has not been helpful because it’s messy sometime*” (Male student tenant, IDI); and “*…we are just coping with it because it’s not really good to share toilet with other families, for me it’s the accommodation’s convenient location that still keeps my household here*” (Female tenant, IDI).

Almost all users saw nothing wrong with sharing compound toilets, especially if the toilets were collectively and properly used and maintained. However, some challenges were reported in addition to those reported earlier:

- The burden of always fetching water to flush a water closet.
- Poor toilet user behaviour—including failure to flush toilet and standing/squatting on toilet seat instead of sitting.
- Some quarrels and conflict among toilet users and cleaners due to failure to clean and/or use toilet properly as expected.
- Frequent desludging with high cost and the failure of some users to pay their contributions.

These challenges are similar to those identified by other studies, which emphasised issues such as water unavailability, poor maintenance, toilet misuse or abuse due to poor behaviour, lack of cleaning materials, as well as conflicts or quarrels—physical fights, and exchange of insults, especially among women when users soiled facilities and/or children dirtied toilets [6,49].

### 3.3. Hygienic Practices around Shared Sanitation

All compound toilets users have arranged that women users clean the facilities, mostly on a daily basis and in turns. These women belong to households that use the toilets. A household without a female is exempted from the cleaning schedules and this is a common practice among all participants. This arrangement stems from culture and local norms that women are responsible for hygiene and sanitation chores and issues in the home, as was reported in similar studies in Kenya [6,50].

“*…whoever is your turn to sweep the compound is also supposed to clean the toilet and that’s the arrangement in my compound.*”

(Two landladies, FGD)

Similar comments on male exemption from cleaning toilets were echoed among all participant groups and also depicted a common perception as conveyed by the quote below.

“*…aaaah, it is only a wicked landlord that will tell a man to clean and scrub toilets, and here in Ayigya I have never heard anything like that!*”

(Landlady, FGD)

However, there were mixed reactions to the involvement of women of landlords’ households in the toilet cleaning chore. Some tenants and landlords claimed that all women, including those of the property owner’s household, were involved in the cleaning as users and residents, while others reported the contrary. A participant had this to say “*… the wife and daughter of the landlord also clean it and not only tenants to avoid conflict from perceived cheating*” (Male, landlords mixed FGD). The statement was supported by another that “*…for us those who use it clean it, everyone sweeps his/her private area, but the cleaning of drains, toilet and bathroom is done according to an agreed verbal duty roster among cleaners.*” (Landlady, mixed FGD). However, a landlady (same mixed FGD) indicated to the contrary, “*…for my house only tenants do the cleaning but when the toilet is full everybody contributes money for desludging.*”

On resources, the commonly used toilet cleaning materials were broom, brush, detergents, water and sometimes disinfectants. These materials were provided by households involved in cleaning, with the exception of a participant who claimed that the landlady of her compound provides the cleaning materials under the condition that the landlady’s household is exempted from the toilet cleaning chores.

### 3.4. Definition of “High-Quality” Shared Toilet Facilities

Study participants reported key considerations that could improve the quality of compound shared toilet facilities. These considerations listed below are also key characteristics perceived to define “high-quality” shared toilets (their ideal shared toilets) among users:

A “high-quality” shared toilet is:

- Always clean and neat for any person ready to use the toilet.
- Devoid of poor user behaviour, such as spitting on the floor, smearing toilet seats and floors with faeces, spilling of water and urine on the floors and seats etc. (“*… toilet is clean, neat, free from odour, bin for used anal cleansing materials emptied every day, toilet bowl/seat is always clean.*” Female tenant, IDI).
- Without any offensive odour (“*…we must also apply chemicals to control odour.*” Landlady, FGD).
- Less crowded with fewer users per facility.
- Timely desludging according to schedules (“*…To me the toilet must be desludged every three months because the pit gets full quickly.*” Landlady, FGD).
- One for which affordable desludging services are available.
- An upgraded toilet facility, such as a water closet with tiled privy rooms (walls and floors).
- Shared among people in the same compound (“*…we tenants do not like sharing our toilets with outsiders because they abuse the use of the toilet—spitting, spilling urine and water etc.*” A male tenant, IDI).
- One that is rehabilitated if it existed as an old facility to enhance cleaning.
- With guided rules enforced and complied by all users.

These descriptive qualities were not different in terms of male and female perspectives, or for tenants and landlords. Neither male or female participants attached any importance to gender-separated toilet facilities except in terms of concerns raised about overcrowding of users. Participants expressed a desire for common high-quality toilet features probably because a “high quality” shared toilet is preferred by all toilet users irrespective of tenancy and gender.

Participants’ perception of “high-quality” shared sanitation, as listed above, suggests concern for collective positive behaviours towards cleanliness, and operations and maintenance culture necessary for compound toilets to become functional [6,9]. Because unhygienic toilets are associated with psychosocial distress and negative health outcomes, improved user behaviour is critical for compound shared sanitation [45].

### 3.5. Understanding Behaviour Change Needs for Improved or High-Quality Shared Toilets

Based on the BCW framework, some behavioural attributes that needed change have been identified (Table 3). These are largely focused on, but not limited to, the synthesis of challenges, benefits, and existing practices associated with participants’ shared toilets. Since there were no major claims against user groups (tenants, landlords, men and women) except children about poor behaviour, it is expedient to make a collective assessment of the emerging unwanted behaviour patterns.

The findings (Table 3) demonstrate the existence of certain behavioural barriers to quality shared toilets, including the incapability of children to use the toilet appropriately, non-written toilet cleaning roster, inadequate toilet facility provision by landlords, and toilet abuses. These barriers would require employing the recommended intervention functions and policy categories postulated by BCW proponents [51]. For the aspects of capabilities, interventions may include educating all users, including children, on the importance of a clean shared toilet; instituting toilet use restrictions for younger children, in particular, demanding parental support; sharing examples of a high-quality compound shared toilet with full benefits in settings similar to their context using audio visuals, including short documentaries (modelling); and supporting user groups to have a written toilet cleaning duty roster instead of relying on memorisation of oral agreements. Meanwhile, these interventions will need policy support, such as all-inclusive (women, men, children, and physically challenged users) toilet design guidelines (technology types and their specifications for various conditions, including number of users), and regulations (e.g., local assembly sanitation by-laws) regarding compound shared toilets. For instance, sanitation by-laws with an effective enforcement regime may achieve the expected high-quality household toilets, even in low-income settlements [38,52].

On the opportunity dimension, interventions should involve joint use of BCW functions of education, coercion, persuasion and incentivisation [53]: to improve the user–toilet ratio to three–five households per facility by landlords or compound owners (to reduce crowding), and for toilet users (landlords and tenants alike) to promptly fix toilet operation and maintenance issues. These interventions will equally need policy categories—legislation, communication, environmental and social planning—that target the barriers identified. For instance, making or changing laws to compel landlords to provide enough compound toilet facilities for occupants (e.g., at most three households per compound toilet), and appropriate communication and education to effectively demystify the taboo of male cleaning of toilets.

The applicable motivational functions for intervention could rely on (1) enablement created by existing agreed cleaning arrangements, and users’ high acceptability of shared toilets; and (2) training all user groups to impart specific skills, such as writing down daily toilet cleaning schedules [45], and to stimulate adoption of locally initiated sanctions agreed among compound toilet users against uncooperative and unruly behaviour (coercion). In addition, regulators (local government) should institute conflict resolution mechanisms and encourage shared toilet users to seek support in matters of conflicts, assaults and quarrels regarding toilet use and management [6]. Furthermore, the regulator should support the provision of appropriate and affordable services to ease the cost burden on desludging and other operation and maintenance services essential for improved compound shared toilets.

## 4. Conclusions

This study has identified key barriers and opportunities associated with proper management of shared sanitation in low-income urban settings, as well as the indicators users consider for improving the quality of shared sanitation (to the standard of the ideal shared toilet). Users are benefiting from their shared sanitation and their perception is instructive towards improving toilet use behaviour and management practices. The compound toilets are overcrowded, and the average crowding is higher than the recommended three households (for a maximum number of users of 20 persons) per toilet facility. Barriers that need significant attention to improve shared sanitation are overcrowding and long queues, particularly where more children are co-users; poor user behaviours from children and some adults; quarrels and conflicts among users and facility cleaners; and the high cost associated with frequent desludging of toilet facilities. The interventions may include effective policies and incentives that target minimising crowding of toilet users (should be defined in regulations), and improving user behaviour in order to offer at least the needed basic sanitation service level. User behaviour change interventions should target, as a minimum, the specific perceived indicators of “high-quality” shared sanitation, such as toilets that are “always clean, without odour, devoid of bad user behaviour and practices, and desludged whenever necessary”.

## Figures and Tables

**Table 1:** Basic profile of participants from the in-depth interviews (IDIs).

Basic Profile	Responses/Distribution	
	Tenants (*n* = 20)	Landlords (*n* = 22)
Participants	Male (5) and Female (15)	Male (8) and Female (14)
Landlord resident	Yes (8 of 20)	Yes (22)
Compound toilet	Landlord-tenant separated (3 of 20) Gender-separated (None)	Landlord-tenant separated (none) Gender-separated (1 of 22)
Number of years spent in compound	3 months to 20 years	8 months to 20 years
Number of households sharing toilet	2–19 households	5–12 households
Number of people sharing toilet	4 to 29 people ~15 people per facility	10 to 25 people ~17 per facility

**Table 2:** Key benefits tenants and landlords derive from using compound shared toilet facilities (IDIs).

Tenants (Number of Times Mentioned)	Landlords (Number of Times Mentioned)	Some Reference Quotes from Participants
Accessible at any time including night hours and emergencies (**20**)	Accessible at any time including night hours and emergencies (**15**)	“*… it has helped compared to my previous accommodation. We can use this toilet at any time unlike public toilets which are mostly locked at midnight.*” (A male tenant, IDI)
Provide convenient access (**10**)	Provide convenient access (**11**)	“*…saves time, within convenient reach, risk of long distance and crossing roads to public toilet averted.*” (Female tenant, IDI) “*…Oh, yes, because you may easily contract diseases from public toilets, implying consequences including hospital bills.*” (Landlord, IDI)
Time savings (toilet within compound, long distance to public toilets avoided) (**9**)	Time savings (toilet within compound, long distance to public toilets avoided) (**9**)	
Provides safety and security than going outside to use public toilets (**4**)	Provides safety and security than going outside to defecate (e.g., use public toilets and open defecation) (**6**)	
Cost savings from using public toilets (**4**)	Cost savings from using public toilets (**6**)	“*…it is cheaper to use compound toilet than public toilet which is pay-as-you-use every day. After all, sometimes for 5 years no desludging cost is incurred on the septic tank or pit of the compound toilet.*” (Landlord, IDI) “*…in fact, it has helped my finances. I do not have to pay to use a public toilet every day.*” (Female tenant, IDI)
-	Controls open defecation and spread of diseases and infections (**2**)	“*Yes, it has been very helpful because we don’t defecate in polythene bags and throw them around which can cause many diseases.*” (Landlord, IDI)
-	Public toilet has long queues (**2**)	“*…we have locked our own so whoever wants to use the facility just goes for the key and use it, there are no queues.*” (Landlord, IDI)
Brings joy and happiness, confers dignity and pride (**1**)	Brings joy and happiness, confers dignity and pride (**1**)	“*…a house without a compound toilet brings unhappiness, shame, embarrassment when your visitors have to find toilet outside to use*” (Female tenant, IDI)

**Table 3:** Analyses of emerging behaviour patterns using the COM-B system of the Behaviour Change Wheel framework.

COM-B Conditions	Aspects	General Observations	Barriers to Positive Behaviour
Capabilities	Physical: physical skills and strength	Users have skills and strength to clean toilet themselves using female members. Complaints about children’s usage of compound toilets.	Children are unable to use toilet properly due to unsuitable design. Lack of written down toilet cleaning duty roster.
	Psychological: resources, ability, skills, knowledge, capacity for understanding	Users appreciate the importance of cleaning toilets. Children are partly blamed for long waiting time and poor toilet use habits. Cleaning arrangements exist as verbal agreement. Some female users unable to use toilet due to poor sanitary conditions.	Lack of users’ full understanding of the need for cleaned toilet. Some users may delay and/or be unwilling to pay for operation and maintenance (O&M). Children lack ability to appreciate and use toilets properly like some adults.
Opportunities	Physical environment: resources, locations, physical barriers, etc	Users provide necessary cleaning materials. At least a toilet facility is available to users. Toilets are overcrowded with users. Compound toilets are easily accessible than public toilets.	Inadequate toilet facilities. Toilets with unfixed maintenance issues are barriers to cleanliness. Lack of water supply for flushing wet toilet systems.
	Social environment: concepts available in language, exposure to ideas, etc.	Some self-esteem and social prestige attached to access to compound toilets over public toilet.	Males cleaning toilet is considered a “taboo”.
Motivation	Reflective: involving goals, self-conscious planning, analysis and decision-making	Users have goals of keeping toilet clean, neat and healthy. Personal efforts and decisions to keep toilet clean after usage. Users know and appreciate benefits from compound shared toilets. User perception that compound toilets are safer and secure than public toilets.	Cleaning arrangements must be translated into written duty roster. Occupants decision to use public toilets. Parents need to help and teach children good toilet use habits.
	Automatic: processes involving emotional reactions, drives, and habits	Some users are passionate about keeping toilets clean and neat. Compound shared toilets are convenient than public toilets.	Some users disregard for decency and poor hygiene habits—toilet abuses. Some users cannot use dirty toilets. Habits of rejecting corrections and overreaction with quarrels.

## Supplementary Materials

The following are available online at https://www.mdpi.com/1660-4601/17/12/4528/s1, Focus group discussion and in-depth interview guides.
