# Supplementary material for: Understanding the Barriers and Opportunities for Effective Management of Shared Sanitation in Low-Income Settlements—The Case of Kumasi, Ghana
Source: Int J Environ Res Public Health. 2020 Jun 23;17(12):4528. doi: 10.3390/ijerph17124528 (PMC7345014; doi:10.3390/ijerph17124528)
Supplement: Supplementary file 1 [file ijerph-17-04528-s001.pdf]

SHARED SANITATION INTERVIEW GUIDE - GHANA

Note: Before beginning the interview, give the necessary information about the study and obtain consent from the respondent.

Find out the respondent's tenure status (E.g. is the respondent a landlord or a tenant) and the type of compound he lives in (E.g. a compound with the landlord living in the compound, a compound with tenants only, a compound with families, a compound with a caretaker, etc).

Suburb/community______________________________

1. Status of respondent – A) landlord B) tenant
2. Does the landlord live with the tenants in the same compound? A) Yes, B) No
3. Who you do you share the toilet with? - *all people who use the toilet (within the compound and also if outside people use it – probe*)
4. When did you begin sharing? *(since when has the respondent been sharing toilet with other households)*
5. What makes you share the toilet? - *what conditions or factors resulted in the respondent sharing toilets with other household – probe for more reasons*
6. What makes you <u>continue</u> sharing the toilet you currently use? – *why (what reasons) have made the respondent not stop sharing toilet with other households?*
7. What were you using before you began sharing the toilet you currently use? – *what was the respondent's experience regarding toilet use and practices before the toilet he/she is currently using*
8. How has the toilet been helpful to you? – *how has the respondent benefited from the current toilet he/she is sharing with other households. Please probe further.*
9. What prevents you or others from using the toilet? – *first find out whether some people on the compound are not using toilet, are there some conditions/factors which can prevent someone from using the toilet, or why can't some people use the toilet if there are some people on the compound who do not use the toilet*
10. What do you need to access the toilet? - *are there some conditions that people should satisfy before they can be allowed to use the toilet? Probe for all possible reasons*
11. What payment do you need to make before using the toilet? – *are there any payment requirements to use the toilet –do some people have to pay before they are allowed to use the toilets? - this applies to all users*
12. What type of toilet is the respondent sharing with others? – *please note the toilet type and provide other descriptions especially if you are not sure of the toilet type*
13. How many people use the toilet? – *find out about how many toilets are on the compound (the number of toilet rooms/cubicles), how households (families) use the toilet, and how many people in all use the toilet facility*

14. How do other users (apart from compound/plot members) get access to use the toilet? *(Probe for agreement with landlords, getting permission, getting a key, paying, etc.)*

15. What do you do to keep off outsiders users from using the toilet? – *probe for more reasons*

16. How would you describe the cleanliness of your toilet? *(probe to understand all the qualities/factors respondents use to describe a toilet which is clean or of high quality)*

17. Who cleans the toilet? – *get details on who is involved in the cleaning of the toilet (all users, tenants, landlords, outsiders, etc.)*

18. How often is cleaning done? – get details on how often cleaning of the toilet is done – several times a day, daily, 2 times weekly, etc.

19. What is the arrangement for cleaning? – *do they have any cleaning arrangements (per households on a daily basis or which frequency), are these arrangements written and pasted somewhere or they are verbal agreement, etc*

20. What is used for cleaning? - *probe to know all the materials used for cleaning of the toilets*

21. What would you say about the cleaning of the toilet? – *do you face challenges with the current cleaning arrangement. What kind of challenges and how are the challenges addressed?*

22. What do you do when the toilet is not cleaned? – *do you have some kind of penalties to those who fail to clean the toilet when it is their turn? If yes, what kind of penalties or punishment and it is often effective?*

23. What do you think can be done to improve the cleanliness of the toilet? - *based on the respondent experience in using shared toilet, what does he/she think can be done to improve cleanliness of the toilets on compound houses – probe for more suggestions*

24. What arrangement is in place to ensure that toilet is kept clean?
    - *Probe*: What happens when this plan is not followed?

25. Who is in charge of maintenance of the toilet? - *what happens if something is not working well on the toilet and need to be repaired or replaced? Who is responsible for this?*

26. How do users contribute to maintenance of the toilet? – *do users contribute for toilet maintenance including desluding? How is this often done?*

27. What is done to ensure that all users contribute to maintenance of the toilet? – *how do you ensure that everybody contributes to toilet maintenance successfully or do you face challenges – if challenges exist, what kind of challenges?*

28. What challenges have you experienced in sharing the toilet? - *probe to know all challenges respondents face when sharing the toilets with other households*

29. What recommendations would you give to improve the practice of sharing toilets? - *based on the respondents experience, what measures does he/she think can be adopted to improve the practice of sharing toilets with other households or to avoid some of the challenges mentioned, or to improve the quality of their shared toilets?*

FOCUS GROUP DISCUSSION GUIDE WITH LANDLORDS

The meeting begins with an ice breaking activity-perhaps a round of introduction.

1. Please describe how you live in your residences-Do you all live with your tenants on the same compound?
2. Do you have toilets on your compounds?
3. Who do you use/share the toilets with?
4. Do you share the toilets with your tenants?
5. How many people share the toilet in your compounds?
6. How do other users (apart from compound/plot members) get access to use the toilet? (Probe for agreement with landlords, getting permission, getting a key, paying, etc.)
7. Are you comfortable with the others using your toilet? Why?
8. What do you do to keep off outsiders users from using the toilet?
9. How would you describe the cleanliness of your toilet?
10. Who cleans the toilet?
11. How often is cleaning done?
12. What is used for cleaning?
13. What do you do when the toilet is not cleaned?
14. What is your role/responsibility in the maintenance of the toilets? And what is the tenant's role/responsibility?
15. What challenges have you experienced in sharing toilets in your compounds?
16. What can be done to improve the cleanliness/quality of the toilets in your compounds and in community as a whole?
17. What strategies do you think can help in keeping the toilets clean?

FOCUS GROUP DISCUSSION GUIDE WITH TENANTS - GHANA

The meeting begins with an ice breaking activity-perhaps a round of introduction.

1. Please describe how you live in your residences-Do you all live with your landlords or caretakers on the same compound?
2. Do you have toilets on your compounds?
3. Who do you use/share the toilets with?
4. Do you share the toilets with the landlords and caretakers?
5. How many people share the toilet in your compounds?
6. How do other users (apart from compound/plot members) get access to use the toilet? (Probe for agreement with landlords, getting permission, getting a key, paying, etc.)
7. Are you comfortable with the others using your toilet? Why?
8. What do you do to keep off outsiders users from using the toilet?
9. How would you describe the cleanliness of your toilet?
10. Who cleans the toilet?
11. How often is cleaning done?
12. What is used for cleaning?
13. What do you do when the toilet is not cleaned?
14. What is your role/responsibility in the maintenance of the toilets? And what is the landlord's/caretaker's role/responsibility?
15. What challenges have you experienced in sharing toilets in your compounds?
16. What can be done to improve the cleanliness/quality of the toilets in your compounds and in your community as a whole?
17. What strategies do you think can help in keeping the toilets clean?
